# Perennial Ryegrass Contains Gluten-Like Proteins That Could Contaminate Cereal Crops

**DOI:** 10.3389/fnut.2021.708122

**Published:** 2021-07-28

**Authors:** Sophia Escobar-Correas, James A. Broadbent, Alicja Andraszek, Sally Stockwell, Crispin A. Howitt, Angéla Juhász, Michelle L. Colgrave

**Affiliations:** ^1^CSIRO Agriculture and Food, St. Lucia, QLD, Australia; ^2^Australian Research Council Centre of Excellence for Innovations in Peptide and Protein Science, School of Science, Edith Cowan University, Joondalup, WA, Australia; ^3^CSIRO Agriculture and Food, Canberra, ACT, Australia

**Keywords:** wild grass, cereal, ryegrass, gluten, wheat, proteomics, LC-MS/MS

## Abstract

**Background:** To ensure safe consumption of gluten-free products, there is a need to understand all sources of unintentional contamination with gluten in the food chain. In this study, ryegrass (*Lolium perenne*), a common weed infesting cereal crop, is analysed as a potential source of gluten-like peptide contamination.

**Materials and Methods:** Ten ryegrass cultivars were analysed using shotgun proteomics for the presence of proteins from the prolamin superfamily. A relative quantitative assay was developed to detect ryegrass gluten-like peptides in comparison with those found in 10 common wheat cultivars.

**Results:** A total of 19 protein accessions were found across 10 cultivars of ryegrass for the protein families of PF00234-Tryp_alpha_amyl, PF13016-Gliadin, and PF03157-Glutenin_HMW. Protein and peptide homology searches revealed that gliadin-like peptides were similar to avenin and gamma-gliadin peptides. A total of 20 peptides, characteristic of prolamin superfamily proteins, were selected for liquid chromatography mass spectrometry (LC-MS) with multiple reaction monitoring (MRM). Only two of the monitored peptides were detected with high abundance in wheat, and all others were detected in ryegrass. Glutenin and alpha-amylase/trypsin inhibitor peptides were reported for the first time in ryegrass and were noted to be conserved across the Poaceae family.

**Conclusion:** A suite of gluten-like peptides were identified using proteomics that showed consistent abundance across ryegrass cultivars but were not detected in wheat cultivars. These peptides will be useful for differentiating wheat gluten contamination from ryegrass gluten contamination.

## Introduction

Gluten proteins are the most abundant proteins found in commercial cereal grains, including wheat, barley, rye and oats (1). Consumption of these proteins will trigger gluten-related disorders (GRD) in ~100 million people globally (2, 3). At least six GRDs (4) have been described; these may be autoimmune, allergic, or neither and are caused by a mix of environmental and genetic factors (4, 5). Coeliac disease (CD) is the most recognised GRD, which is currently diagnosed based on serology and small intestinal biopsies and is estimated in 0.7–1.4% of the global population (2). Affected individuals are genetically susceptible to generate an autoimmune inflammatory response in the small intestine when exposed to gluten proteins. Long-term exposure results in chronic intestinal inflammation and villi degradation for these individuals (6).

The only treatment for GRDs is to avoid the intake of trigger proteins, i.e., commit to a life-long gluten-free diet (GF-diet), although this is far from simple or easy. Complications arise through the difficulty in avoiding gluten-containing additives, inadvertent gluten intake, and food contamination. Gluten is frequently included in nongrain-based items such as sausages, salad dressing, imitation meats, and even some medications (7, 8). Therefore, the GF-diet mainly consists of fruits, vegetables, legumes, meat, seafood, nuts, dairy and bakery products, which include GF cereals or pseudocereals, such as rice, corn, quinoa and millet (9, 10). Nevertheless, these products may contain hidden gluten due to unintentional contamination through the food supply chain.

Cross-contamination within the food supply chain can happen at different stages, such as production, milling, export and retail. There are multiple possibilities from the moment the grain is grown until the GF-flour is packed, including agricultural co-mingling through crop rotation, storage and transport. Some studies provide examples in the contamination of GF-oats with gluten-containing cereals during harvest (11–13). One further aspect in this regard concerns the contamination of GF products due to weeds growing in the field, a topic that is under-researched. Weed management in crops is a challenging task. Farmers spend thousands of dollars each year in an effort to control weed invasion; however, this outcome is not always completely accomplished, and farmers must deal with weed seed contamination, which can become a serious problem for GF-cereals and other crops that are supposed to be free from gluten, such as pulses (14).

One of the most common weed seeds found in cereal samples is ryegrass (genus *Lolium*) (14). This grass is the most common weed infesting cereal grain fields in Australia and has small dense seeds that are difficult to eliminate during automated grain cleaning (14, 15). Ryegrass belongs to the same Poaceae grass family as wheat and other gluten-containing crops, wherein the storage proteins primarily comprise gluten-like proteins. Ryegrass has been subjected to Western blotting followed by liquid chromatography mass spectrometry/mass spectrometry (LC-MS/MS) analysis, demonstrating that it contains proteins with structural similarity with gluten proteins (15).

Herein, a combination of discovery and targeted proteomic analyses were undertaken to confirm the presence of gluten-like proteins in ryegrass cultivars. A targeted LC-MS/MS assay was developed and used to assess relative levels of target peptides across 10 ryegrass cultivars and 10 wheat cultivars to identify the differences in peptide abundance patterns and to identify possible ryegrass-specific peptide markers.

This study investigates whether gluten contamination can potentially originate from sources other than traditional cereal grains, such as field contaminants. Understanding the possible origins of gluten contamination and establishing identification and quantification methods for these new protein species could help to provide a more accurate characterisation of food and assure food safety for the population affected by GRDs.

## Materials and Methods

### Sample Material

Grain samples from 10 ryegrass and 10 wheat cultivars were obtained (Supplementary Table 1) from the Australian pasture collection and Australian Winter Cereals Collection (Tamworth, Australia). All samples were manually inspected to exclude foreign seed contamination. Flour samples were obtained by milling with a Metefem Hungarian Mill (model FQD2000, Hungary).

### Protein Extraction and Digestion

Methods were performed according to the study of Bose et al., with minor changes (16). Flour (20 mg) was weighed (four replicates of wheat and three replicates of ryegrass) and mixed with 200 μL of 55% isopropanol and 2% dithiothreitol (IPA/DTT). Samples were vortexed and sonicated for 5 min and then incubated on a thermo heating mixer block (Thermo Scientific) at 300 rpm at 65°C for 45 min. The mixtures were centrifuged for 15 min at 20,800 × g, and the supernatants were transferred to Protein LoBind Tubes (Eppendorf). The protein content was quantified *via* Bradford assay, and 200 μg of protein was loaded onto 10-kDa molecular weight cut-off (MWCO) filters (Millipore, Sydney, Australia). Aliquots were adjusted to 200 μL with UA buffer (8 M urea in 0.1 M Tris-HCl, pH 8). The filter content was washed two times with 200 μL UA buffer followed by centrifugation at 20,800 × g for 15 min at room temperature (RT).

Alkylation of cysteines was performed by the addition of 100 μL of 50 mM iodoacetamide (in UA buffer) and incubation in the dark at RT, with 300 rpm shaking for 20 min. Samples were centrifuged for 15 min at 20,800 × g and then washed with 200 μL UA buffer and centrifuged again, as previously described. Flowthrough was discarded and the buffer was changed to 50 mM AmBic (ammonium bicarbonate, pH 8.0) by washing the filters two times with 200 μL of this digestion buffer followed by centrifugation. Filters were transferred to fresh collection tubes. Protein digestion was achieved by adding 200 μL of trypsin (Promega, Wisconsin, USA) prepared as 200 μg/mL in 50 mM AmBic (pH 8.0) containing 1 mM CaCl_2_ and incubated overnight in the dark at 37°C with 300 rpm shaking. Sample filters were centrifuged at 20,800 × g for 15 min, followed by two washes with 200 μL of AmBic (pH 8.0) and centrifugation. Filtrates were evaporated to dryness in a Savant SpeedVac concentrator (Thermo Scientific, USA). Peptides were reconstituted in 0.1% formic acid to a protein concentration of 1 μg/μL for LC-MS/MS analysis.

### Mass Spectrometry and Protein Identification

Liquid chromatography mass spectrometry was performed using an Ekspert nanoLC415 (Eksigent, Dublin, CA) coupled to a TripleTOF^®^ 6600 mass spectrometer (SCIEX, Redwood City, CA, USA). The specifications of the acquisition parameters have been previously described (15, 16).

Discovery data was generated for 10 ryegrass cultivars, and the spectra were searched against the Poaceae grass family subset of the UniProt database (version 2021/01) appended with the common repository of adventitious proteins. The database from a higher taxonomic group was used due to poor representation of the *Lolium perenne* proteome (748 nonredundant protein sequences). The UniProt UniRef 100 redundancy reduction was applied to remove Poaceae proteins with 100% sequence identity, leading to a total of 1,953,474 protein sequences.

ProteinPilot v5.0.3 software (SCIEX) encompassing the Paragon and ProGroup algorithms (17) was used to identify peptides, infer proteins, and generate false discovery rate (FDR) reports. Results from discovery analysis were curated using an in-house script (git-hub/Sophia-006^1^) (18). To ensure quality in the identification of gluten proteins and peptides, the following curation parameters were applied: 1% FDR or 99% peptide confidence, requisite tryptic and semi-tryptic peptides, up to two missed cleavages, and variable modifications of glutamine to pyro-glutamic acid, carbamidomethyl cysteine, and oxidation of methionine.

Protein summaries (Supplementary Data 1) were analysed to identify proteins of interest, i.e., gluten proteins. In this regard, a protein family (Pfam) search was performed that encompassed searching for three specific domains: Gliadin (PF13016), Glutenin_HMW (PF03157), and Tryp_alpha_amyl (PF00234). The Pfam search was performed using profile hidden Markov models 3 (HMMER3) (19). Protein and peptide homology searches were performed using the BLAST algorithm and Peptide Search tool available at UniProt^2^, respectively.

Proteins that contained gliadin, glutenin_HMW, and Tryp_alpha_amyl domains were selected for targeted assay development. These protein sequences were gathered into a FASTA file that was used to construct a table of peptide multiple reaction monitoring (MRM) transitions using Skyline Software (MacCoss Lab Software, Washington, USA) (20). MRM transitions were determined for each peptide. Peptides were selected for MRM based on the following criteria: (1) tryptic or semi-tryptic; (2) identified with 95% confidence; (3) unmodified or common modifications only; and (4) high peak signal intensity. Transitions for each selected peptide were prioritised from the acquired discovery data, including precursor ion (Q1) and fragment ion (Q3) m/z, and rolling collision energy (Supplementary Data 2). In total, 20 peptides were selected for MRM experiments. Four semi-tryptic peptides were included; their fully tryptic versions were not included in the final MRM due to insufficient evidence in the discovery data. Three MRM transitions were monitored per peptide based on the intensity and lack of interferences.

Digested peptides were separated with a Shimadzu Nexera UHPLC (Rydalmere, Australia) and analysed with a 6500 QTRAP mass spectrometer (SCIEX), as described previously (15, 16). Relative quantitation was achieved using scheduled MRM scanning experiments with a 60-s detection window for each MRM transition with retention time as determined in preliminary MRM experiments and a 0.6-s cycle time. Peaks were integrated using Skyline, wherein all three transitions were required to coelute at the same retention time (min) with a signal-to-noise ratio (S/N) > 3. The peak areas for the three monitored MRM transitions were summed. The mean, SD, and co-efficient of variation (CV) were calculated for technical replicates for each peptide (Supplementary Data 3). Batch and injection order effects were removed from the data by monitoring external standards interspersed with the unknown samples while retaining the differences between ryegrass and wheat samples.

Graphs were generated in the R statistical computing environment using the ggplot package (21) and Morpheus^3^ (Broad Institute, Cambridge MA, USA).

## Results

### Gluten Protein Identification Yield From Shotgun Proteomics

**Figure 1:**
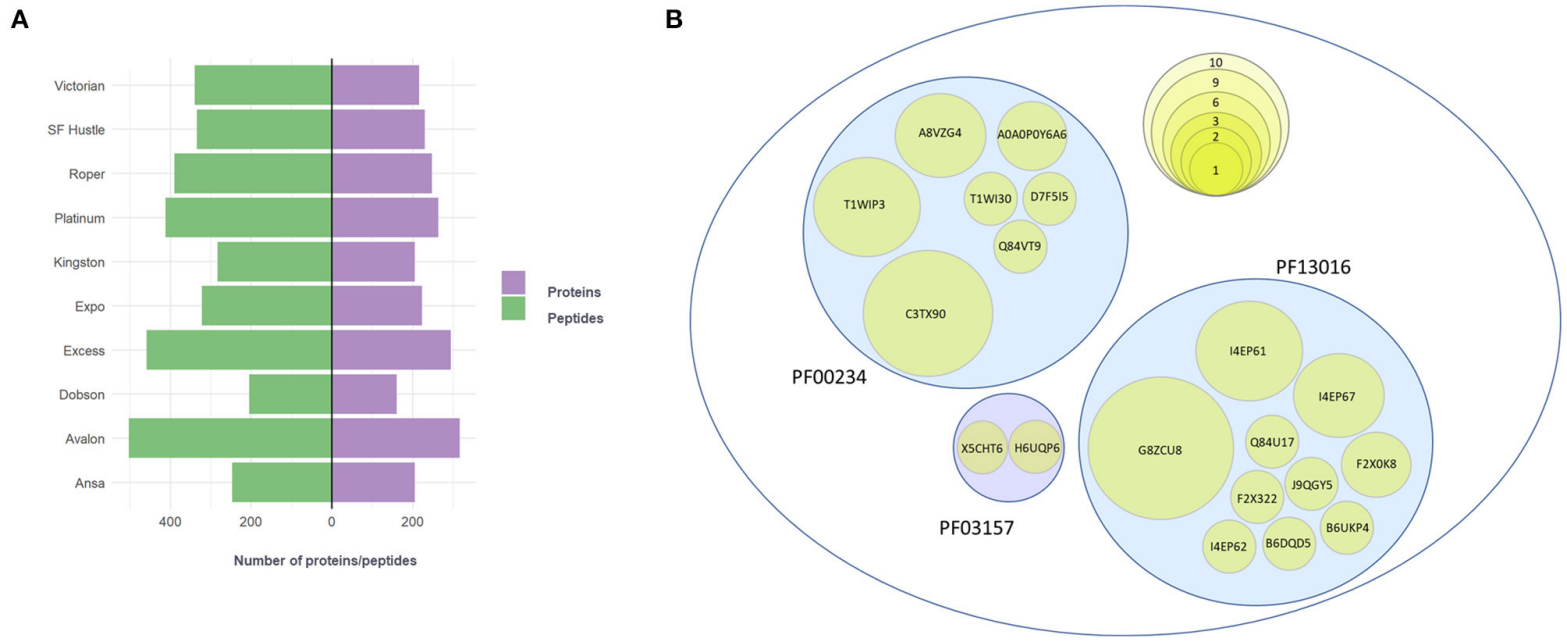
**(A)** Number of proteins (purple) and peptides (green) detected at 1% FDR (99% confidence) for 10 ryegrass cultivars. **(B)** Proteins identified with the protein family search (Pfam) that belong to three specific families: PF00234-Tryp_alpha_amyl, PF13016-Gliadin, and PF03157-Glutenin_HMW. The size of the circle represents the number of identification occurrences among 10 cultivars of ryegrass.

Ten varieties of *L. perenne* were studied (Supplementary Table 1) to confirm the suspected presence of gluten in this species. For the identification of gluten proteins, high-resolution data acquisition and database searching was performed using a UniProt database from a higher taxonomic group belonging to the family Poaceae. The total number of proteins identified with 99% confidence between the 10 varieties of ryegrass varied between 160 and 316 (Figure 1A). Additionally, peptides discovered with tryptic digestion varied between 205 to 503 (Figure 1A).

Next, a protein family domain search was performed for each of the cultivars with the aim to find proteins containing domains characteristic of gliadin, glutenin_HMW, and tryp_alpha_amyl domain families. The results revealed that between three to eight candidate proteins were found for each of the cultivars (Supplementary Data 4). A total of 19 protein accessions were found among the 10 varieties of ryegrass. A table showing positive identification of proteins representing each domain family across the varieties is provided in Supplementary Data 4. For the tryp_alpha_amyl-domain containing protein family (Figure 1B), the candidate protein with the highest number of identification occurrences was UniProt accession C3TX90 (present in nine varieties), followed by proteins T1WIP3 (present in six varieties) and A8VZG4 (present in three varieties). The lowest number of identification occurrences were for A0A0P0Y6A6 (present in two varieties) and for proteins D7F5I5, Q84VT9, and T1WI30 (present in one variety). The gliadin-domain containing protein family (Figure 1B) was represented by the proteins G8ZCU8 (present in all the varieties) and I4EP61 (present in six varieties). Further gliadin-domain containing members were detected between one and three varieties (I4EP57, F2X0K8, I4EP62, J9QGY5, B6DQD5, Q84U17, F2X322, and B6UKP4). The glutenin_HMW-domain containing protein family (Figure 1B) was the family with the lowest representation with two candidate proteins, H6UQP6 and X5CHT6, detected in one ryegrass variety.

Of note, these domains could represent multiple protein types due to the mixed nature of gluten-type proteins, i.e., families which comprise a range of proteins that have the Tryp_alpha_amyl domain or the gliadin-domain are not necessary α-amylase/trypsin inhibitor proteins or gliadins. As such, additional protein and peptide homology searches were performed to determine the full-length homology of these proteins.

Gluten proteins tend to have conserved regions, and further bioinformatic analysis is necessary to determine which peptides could function as peptide markers to differentiate between grass species. As a result, candidate proteins and peptides were subject to searches to identify non-ryegrass orthologues, i.e., to determine the potential for these peptide sequences to be observed in additional species. In consideration of the limitations of mass spectrometric detection, search settings included leucine and isoleucine equivalence (isobaric amino acids) and a requisite C-terminal arginine or lysine (trypsin cleavage site).

Results of the searches are shown in Table 1, and a detailed table specifying the species within the tribe identity match can be found in Supplementary Table 2. Peptides SQILQQSSCQVMR (G8ZCU8), CPAIHSVVQAIILQK (I4EP61), QFLVQQCSPVAEVPFLR (I4EP61), and QQAQFEGMR (I4EP57) were found to belong exclusively to avenins present in oats, while peptides QQCCQQLAQIPQQLR (F2X0K8) and APFASIVASIGGQE (F2X322) belong to gamma-gliadins that are present in wheat. An additional gamma-gliadin peptide, APFASIVAGIGGQYR (B6DQD5), was found in species of the Triticeae tribe.

**Table 1:** Protein and peptide homology search results.

**Peptide sequence**	**Protein accession**	**Protein family** **domain**	**Protein homology** **(from BLAST)**	**Peptide search 100%** **identity match (Tribe)**
SQILQQSSCQVMR	G8ZCU8	PF13016	Avenin	Aveneae
CPAIHSVVQAIILQK	I4EP61	PF13016	Avenin	Aveneae
QFLVQQCSPVAEVPFLR	I4EP61	PF13016	Avenin	Aveneae
AFALQALPAMCDVYVPPHCSVA	I4EP61	PF13016	Avenin	Aveneae
QQAQFEGMR	I4EP57	PF13016	Avenin	Aveneae
QQCCQQLAQIPQQLR	F2X0K8	PF13016	Gamma-gliadin	Triticeae
APFASIVASIGGQE	F2X322	PF13016	Gamma-gliadin	Triticeae
APFASIVAGIGGQYR	B6DQD5	PF13016	Gamma-gliadin	Triticeae
QQCCQQLAQIPEQSR	J9QGY5	PF13016	LMW-glutenin	Triticeae
SQMLQQSSCHVIR	J9QGY5	PF13016	LMW-glutenin	Triticeae
DVSAKCRPVAVSQVAR	X5CHT6	PF03157	HMW-glutenin	Triticeae
ELQESSLEACRQVVDQQLAGR	X5CHT6	PF03157	HMW-glutenin	Triticeae
ELQESSLEACR	X5CHT6	PF03157	HMW-glutenin	Triticeae
QLQCERELQESSLEACR	X5CHT6	PF03157	HMW-glutenin	Triticeae
DGSFYPGEATPPQQLQQR	H6UQP6	PF03157	HMW-glutenin	Triticeae
RCCDELSAIPAYCR	Q84VT9	PF00234	Trypsin inhibitor	Triticeae
LQCVGSQVPEAVLR	T1WIP3	PF00234	Dimeric alpha-amylase inhibitor	Triticeae
EGMEVFPGCR	T1WIP3	PF00234	Dimeric alpha-amylase inhibitor	Triticeae
LLQQQLNPCR	A8VZG4	PF00234	Alpha-gliadin	Triticeae
LTAASVPAVCK	T1WI30	PF00234	Dimeric alpha-amylase inhibitor	Triticeae
TACNCLK	A0A0P0Y6A6	PF00234	Nonspecific lipid-transfer protein	Highly conserved. Families: Poaceae, Fabaceae, Asteraceae
CGVSIPYTISPSIDCSR	A0A0P0Y6A6	PF00234	Nonspecific lipid-transfer protein	Oryzeae
DPYYEQCPMRK	C3TX90	PF00234	Puroindoline-like protein	Brachypodieae
SDLYGPNLQGEVTMLMER	C3TX90	PF00234	Puroindoline-like protein	Brachypodieae
QLSQIAPQCR	D7FSI5	PF00234	Puroindoline, Hordoindoline	Triticeae

*Peptide sequence, protein accession and protein family are specified. Protein homology refers to all protein types where the peptide was found. Peptide search results refer to all tribes where the peptide was found with a 100% identity match*.

Two peptides, QQCCQQLAQIPEQSR and SQMLQQSSCHVIR, from the protein J9QGY5, which is a low molecular weight glutenin (LMW-GS), belong exclusively to the wild grass species *Dasypyrum villosum* of the Triticeae tribe. HMW glutenin (HMW-GS) peptides DVSAKCRPVAVSQVAR, ELQESSLEACRQVVDQQLAGR, and QLQCERELQESSLEACR (X5CHT6) were moderately conserved and found across species of the Triticeae tribe. The peptide DGSFYPGEATPPQQLQQR (H6UQP6) was exclusively found in *Elymus libanoticus*, a species that belongs to the Triticeae tribe.

Peptide EGMEVFPGCR (T1WIP3) was found exclusively in the species *Elymus grandis* (Triticeae); peptide LTAASVPAVCK (T1WI30) was found in several species of the Triticeae tribe. The peptide LLQQQLNPCR (A8VZG4) was found in an α-gliadin sequence of species *Dasypyrum hordeaceum* (Triticeae). For A0A0P0Y6A6, which presented protein homology to a lipid-transfer protein, the conserved peptide TACNCLK was found in species of families Poaceae, Fabaceae, Asteraceae, among others; however, the peptide CGVSIPYTISPSIDCSR was exclusively found in species of rice of tribe Oryzeae. The two peptides DPYYEQCPMRK and SDLYGPNLQGEVTMLMER (C3TX90), from a puroindoline-like protein were found exclusively in *Brachypodium sylvaticum* of tribe *Brachypodieae*. Peptide QLSQIAPQCR (D7FSI5), characteristic of puroindolines and hordoindolines, was present in several species of the Triticeae tribe.

### Gluten-Like Peptide Quantification in Wheat and Ryegrass

Peptides characteristic of gluten-like proteins were selected for LC-MRM-MS analysis. Based on the results obtained from homology searches, 20 peptides from 13 proteins were measured with a relative quantitative analysis (Supplementary Data 5). The selected peptides were measured in 10 cultivars of perennial ryegrass (*L. perenne*) to determine the abundance of gluten candidates. At the same time, these same peptides were measured in 10 wheat varieties to compare the abundance between these two species, aiming to discover peptides that can differentiate ryegrass from wheat.

**Figure 2:**
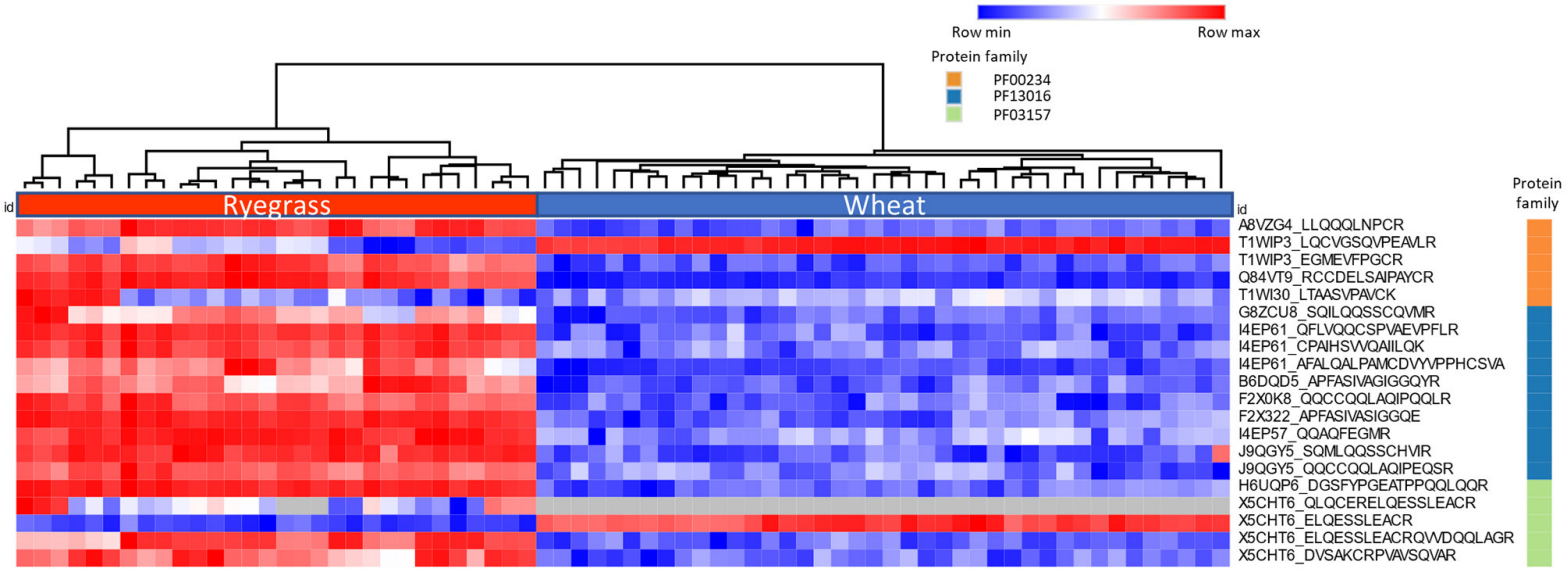
The Heatmap and hierarchical clustering show the relative abundance of the peptides representing two major groups detected across 10 ryegrass cultivars (*n* = 3) and 10 wheat cultivars (*n* = 4). The colour in each cell represents the log (peak area) of each peptide monitored (red = max value, blue = min value, grey = NA). The column to the right indicates the protein family membership for the peptides (orange = PF00234-Tryp_alpha_amyl family, blue = PF13016-Gliadin, green = PF03157-Glutenin_HMW).

The heatmap (Figure 2) shows the logarithmic relative abundance of the measured peptides across 10 ryegrass cultivars (*n* = 3) and 10 wheat cultivars (*n* = 4). The complete Euclidean linkage method was used for hierarchical clustering, which shows clear stratification of ryegrass and wheat. Protein family domain membership for each protein is also specified within the graph. The analysis revealed the peptides measured by LC-MRM-MS in ryegrass cultivars had low abundance or were not detected in wheat.

### Relative Quantitation of Gluten Proteins Across Ten Cultivars of Ryegrass

**Figure 3:**
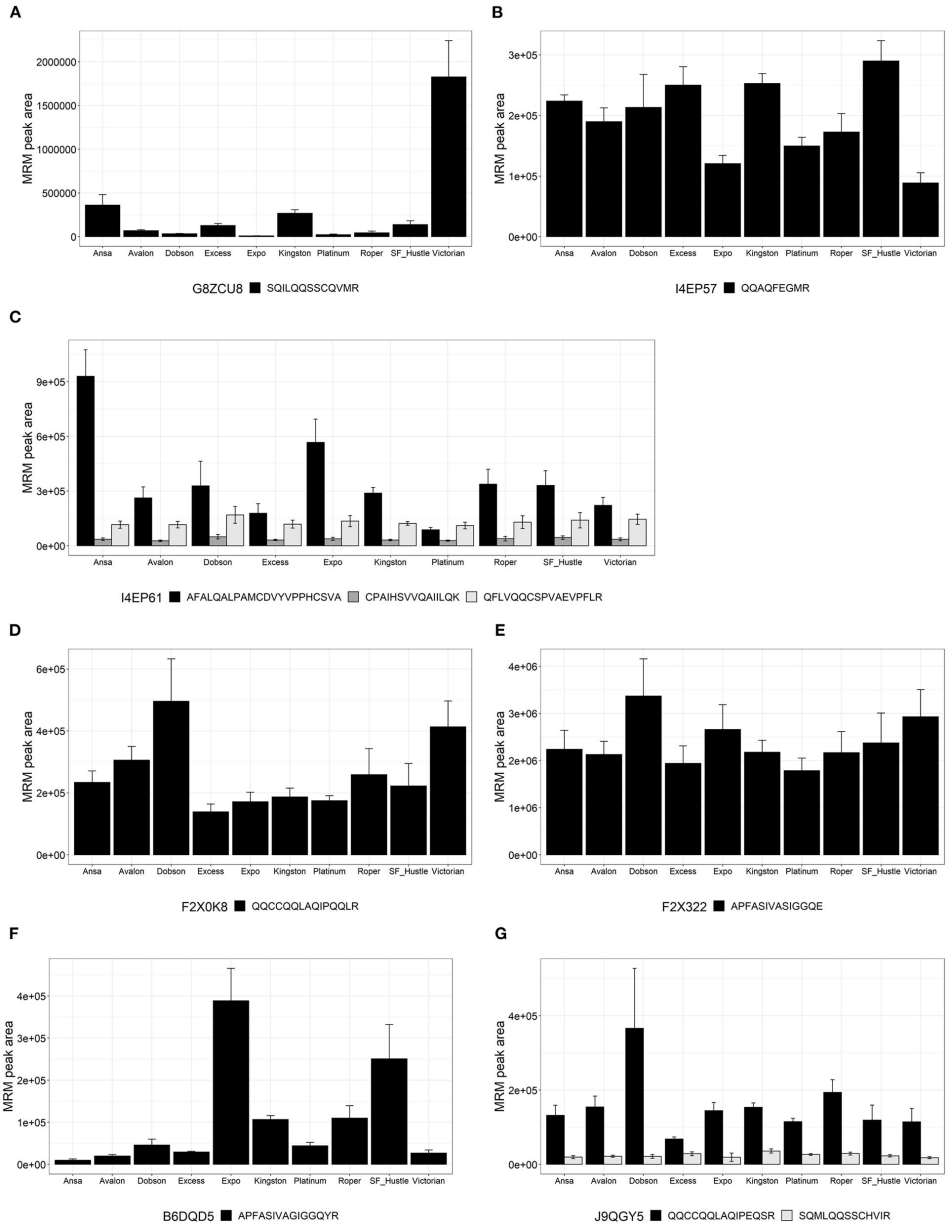
Relative quantitation expressed as multiple reaction monitoring (MRM) peak area for peptides from proteins of the family PF13016 across 10 cultivars of *Lolium perenne*. Data are presented as the mean ± SD (*n* = 3) with one to three peptides from each protein. **(A)** Avenin protein (G8ZCU8); **(B)** Avenin (I4EP57); **(C)** Avenin (I4EP61); **(D)** Gamma-gliadin (F2X0K8); **(E)** Gamma-gliadin (F2X322); **(F)** Gamma-gliadin (B6DQD5); and **(G)** LMW-glutenin (J9QGY5).

**Figure 4:**
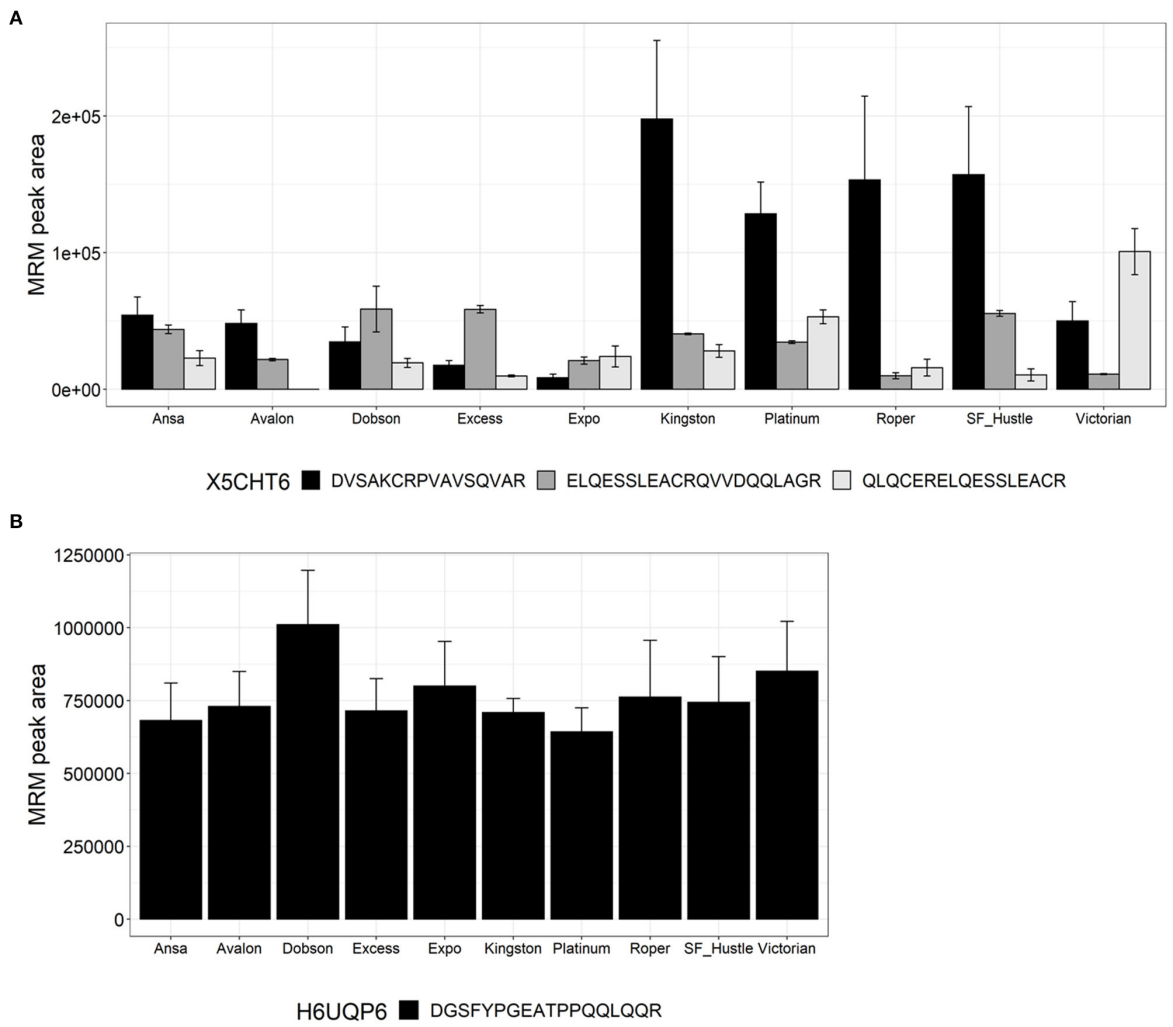
Relative quantitation of glutenin peptides across the 10 varieties of *L. perenne* from proteins of the family PF03157. Data are presented as the mean ± SD (*n* = 3) with one to three peptides from each protein. **(A)** HMW-glutenin (X5CHT6); and **(B)** HMW-glutenin (H6UQP6).

**Figure 5:**
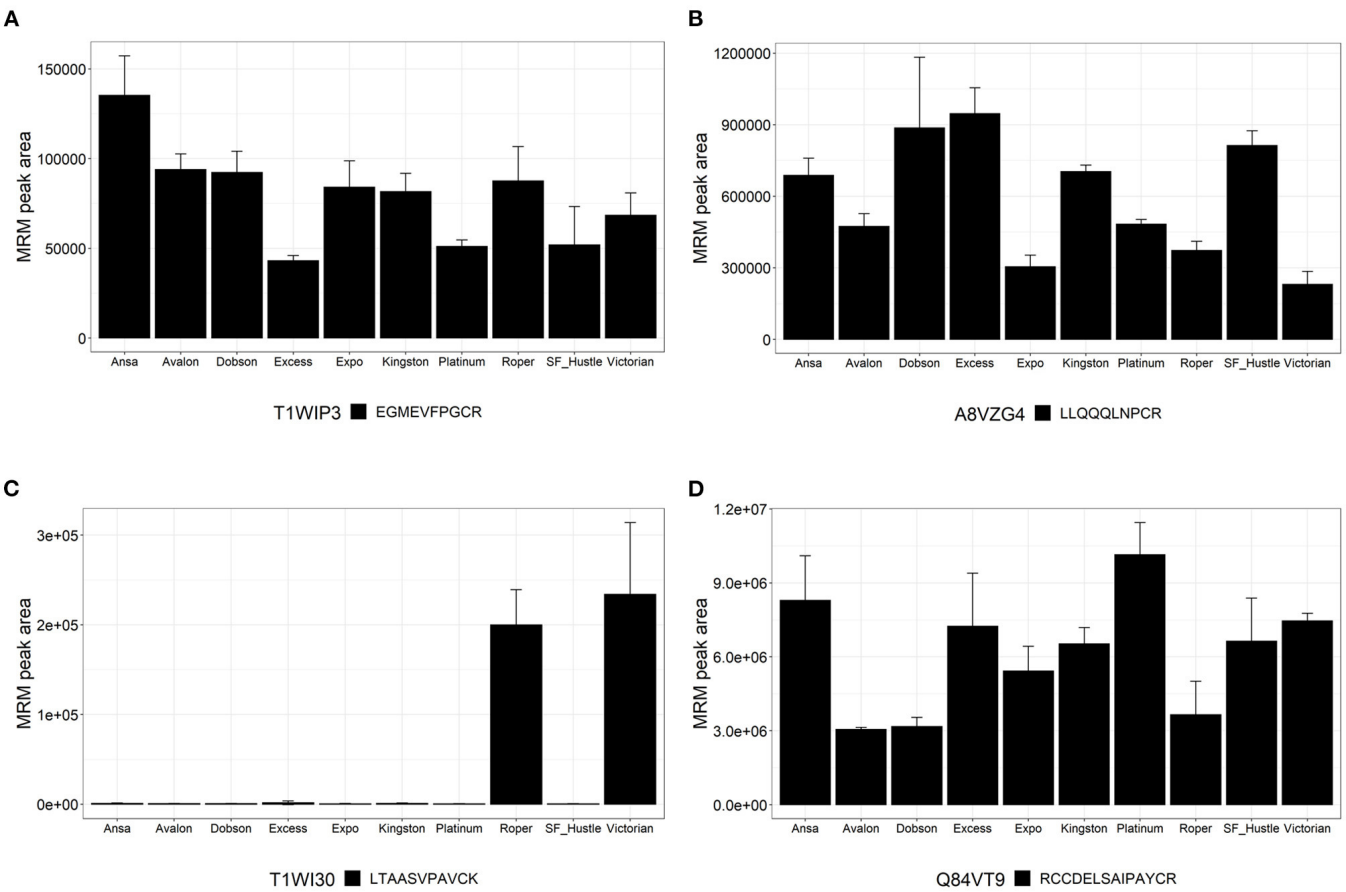
Relative quantitation of peptides across the 10 varieties of *L. perenne* from proteins of the Pfam family PF00234. Graphs show the MRM peak area for each cultivar. Data are presented as the mean ± SD (*n* = 3) with one peptide from each protein. **(A)** Dimeric alpha-amylase inhibitor (T1WIP3); **(B)** Alpha-gliadin (A8VZG4); **(C)** Dimeric alpha-amylase inhibitor (T1WI30); and **(D)** Trypsin inhibitor (Q84VT9).

Except for the peptides mentioned in the section Gluten-like peptide quantification in wheat and ryegrass above, the remaining peptides were only quantified in ryegrass. Their summary MRM peak area results are shown in Figures 3–**5** according to their protein family domain membership. The biological variation between cultivars is presented in Supplementary Data 6.

Results for the gluten-like peptides of family PF13016 Gliadin domain-containing proteins (akin to avenin-like proteins and gamma-gliadins) are shown in Figure 3. Peptide SQILQQSSCQVMR (Figure 3A) from protein G8ZCU8 showed high variability across ryegrass cultivars with the highest abundance in the cultivar Victorian. Peptide QQAQFEGMR (Figure 3B) from protein I4EP57 showed variability among all cultivars of ryegrass with a biological co-efficient of variation (CV) of 32%. Peptide AFALQALPAMCDVYVPPHCSVA (I4EP61) varied across the ryegrass cultivars and showed high values; however, CPAIHSVVQAIILQK and QFLVQQCSPVAEVPFLR (I4EP61) (Figure 3C) showed lower values but were consistently found among all ryegrass cultivars, with a biological CV of <20%. The peptide QQCCQQLAQIPQQLR (Figure 3D) from protein F2X0K8 shows moderate variance across the cultivars, showing higher values for Dobson and Victorian. Peptide APFASIVASIGGQE from F2X322 (Figure 3E) was consistent across all ryegrass cultivars showing a CV of 20%. Peptide APFASIVAGIGGQYR from protein B6DQD5 was variable among ryegrass cultivars and was higher in cultivars Expo and SF Hustle. From protein J9QGY5, two peptides were measured (Figure 3G): QQCCQQLAQIPEQSR was not only more abundant but also more variable across cultivars and was higher in the Dobson cultivar and SQMLQQSSCHVIR was in lower abundance but was consistent (CV <25%) across ryegrass cultivars.

Figure 4 shows the quantitation of peptides from proteins belonging to the family PF03157 HMW-glutenin. Peptides belonging to two proteins, namely X5CHT6 and H6UQP6, were measured. Three peptides were quantified from protein X5CHT6, each showing high variability across the cultivars. Peptide DVSAKCRPVAVSQVAR showed higher abundance in Kingston, Platinum, Roper and SF Hustle; peptide ELQESSLEACRQVVDQQLAGR was highest in abundance in Dobson, Excess and SF Hustle, while peptide QLQCERELQESSLEACR was the highest in Victorian (Figure 4A). The second HMW glutenin, protein H6UQP6, was quantified using peptide DGSFYPGEATPPQQLQQR with low biological variance across cultivars with a CV <15% (Figure 4B).

The detection of peptides from PF00234 Tryp_alpha_amyl domain-containing proteins are shown in Figure 5; these proteins were also shown to have homology to gluten-like proteins (Table 1). One peptide EGMEVFPGCR was observed to be variable across ryegrass cultivars. This peptide is found in protein T1WIP3, a dimeric alpha-amylase inhibitor (Figure 5A). Peptide LLQQQLNPCR was variable across ryegrass cultivars and is present in protein A8VZG4, which is an α-gliadin (Figure 5B). Another peptide LTAASVPAVCK showed variable abundance with high levels in cultivars Roper and Victorian. This peptide is found in protein T1WI30, a dimeric alpha-amylase inhibitor (Figure 5C). Peptide RCCDELSAIPAYCR (detected as protein Q84VT9, a trypsin inhibitor) showed good signal intensity but varied across the ryegrass cultivars (Figure 5D).

## Discussion

Ryegrass (genus *Lolium*) has been identified as one of the most challenging weeds for cropping systems due to its ability to resist herbicides, consequently affecting different farming practises (22). Furthermore, preliminary investigation of ryegrass proteins has revealed its potential as a source of gluten contamination (15, 23, 24). In this regard, an isolation and purification method for the prolamin fraction of ryegrass grains, which was named loliin, was established as early as the 1930s (23). This study preceded the study of Shewry in 1986, who characterised prolamins from different grasses, including *L. perenne*, and established homology of ryegrass γ-prolamins to those from wheat, barley and rye. More recently, Colgrave et al. revealed the reactivity of ryegrass prolamins to the anti-gliadin antibody and identified possible antigenic proteins through LC-MS analysis of gel-separated proteins (15).

Herein, we report the identification of prolamin super-family peptides in ryegrass cultivars and their quantitation when compared with wheat, with the aim to measure differences in peptide abundance and identify potential peptide markers of ryegrass contamination. To this end, grain proteins from 10 cultivars of diploid perennial ryegrass (*L. perenne*) were processed to enrich for seed storage proteins and measured by LC-MS/MS in the search for gluten-like sequences.

Shotgun proteome measurement and database searching were used for the initial discovery of gluten-like protein sequences. The protein search database comprised the public sequences of the Poaceae family proteins from UniProt, a higher taxonomic group for wheat and ryegrass representing diverse genetic variations. Previous proteomics studies in *L. perenne* have used a different database approach where *Brachypodium distachyon* sequences were searched to successfully characterise drought response in the leaves from this grass (25); because of the focus on this compartment, no gluten-like proteins were identified in this study. No other studies besides Colgrave et.al. reported proteome measurement in ryegrass seeds (15).

The discovery strategy herein was complemented by a protein family domain search identifying three to eight gluten-like proteins in each ryegrass cultivar (Supplementary Data 4). This number is lower when compared with the frequency of gluten proteins identified when searching traditional cereal grain data (e.g., wheat, rye, barley) against the same Poaceae subset of the UniProt-KB database, which typically varies between 5 and 47 gluten proteins (26, 27). However, the gluten-like protein sequences in ryegrass may differ from those in wheat and other Poaceae members. Therefore, further investigation is needed to precisely characterise the prolamins in ryegrass. Nevertheless, the strategy to determine gluten-like proteins through detecting the protein family domains for Gliadin, Glutenin_HMW, and Tryp_alpha_amyl revealed 19 protein accessions representing these families, including: 10 gliadin-domain containing proteins, 2 glutenin_HMW domain containing proteins, and 7 Tryp_alpha_amyl domain-containing proteins (Figure 1). Due to a paucity of data (protein sequences) in the *Lolium* subset of the UniProt database (748 nonredundant protein sequences), the identification of gluten-like peptides is likely not exhaustive and has led to the detection of proteins from other species, i.e., orthologous proteins. The implementation of genomic and/or transcriptomic sequencing efforts would allow more ryegrass gluten-like proteins to be discovered.

A targeted MRM assay was developed for specifically measuring peptides found in proteins with homology to gliadins, glutenins, and ATIs. Prior to measuring these peptides, we expected that some targets would be detected at high levels in ryegrass and not in wheat, since target peptides were identified from ryegrass discovery proteomics. The results of this study revealed clear differences between the peptide content in ryegrass and wheat, with peptides predominant in ryegrass, regardless of belonging to the same family of Poaceae.

### Gluten Relative Quantitation and Potential Markers

#### Gliadin and HMW Glutenin Family

In this study, nine peptides were identified from seven protein sequences, characteristic of prolamin proteins, and were measured by LC-MRM-MS. Six of these proteins belong to the Gliadin domain containing protein family and one to the Tryp_alpha_amyl family. Peptide search analysis revealed that five peptides (SQILQQSSCQVMR, CPAIHSVVQAIILQK, QFLVQQCSPVAEVPFLR, AFALQALPAMCDVYVPPHCSVA, and QQAQFEGMR) were primarily detected in avenin proteins (Table 1, Supplementary Table 2). In ryegrass, the peptides detected in all cultivars with consistent levels were QFLVQQCSPVAEVPFLR and CPAIHSVVQAIILQK (Figure 3C). These peptides are not found in wheat, barley, or rye; therefore, these peptides are possible markers for differentiating ryegrass contamination from traditional gluten-containing grains. However, they are also common to *Avena sp*. and thus will not discriminate ryegrass from oats (Supplementary Table 2). Another peptide, LLQQQLNPCR (Figure 5B), from protein A8VZG4, a Tryp_alpha_amyl domain containing protein family member, was present in an α-gliadin of the species *D. hordeaceum* of the Triticeae tribe. However, this peptide may not be an ideal peptide marker due to its lack of uniform signal. Three peptides (QQCCQQLAQIPQQLR, APFASIVASIGGQE, and APFASIVAGIGGQYR, Figures 3D–F) were matched back to γ-gliadin sequences from species of the Triticeae tribe; interestingly, though these specific peptides were not detected in the wheat cultivars tested. These peptides showed variable abundance across the ryegrass cultivars, and although not detected in the wheat cultivars examined herein, these peptides will not make ideal markers for ryegrass contamination due to their presence in known wheat γ-gliadin sequences.

The nomenclature of prolamin super-family proteins is diverse depending on the cereal of origin. In wheat, these proteins are gliadins; in rye, they are secalins; in barley, hordeins, and oats, they are avenins. The phylogenetic relationship between prolamin proteins from different species has been demonstrated with homology comparisons between avenin sequences and α- and γ-gliadins from wheat, B-hordeins from barley, and γ-secalins from rye (28–30). Herein, we showed that ryegrass has gliadin-like peptides that share a certain level of similarity to avenins and γ-gliadins. Moreover, this agrees with a comparative genomic study that revealed conserved genetic maps in terms of orthology and collinearity in the lineage of the Triticeae (wheat), Aveneae (oat), and Poeae tribes (ryegrass) (31–33), although phylogeny studies place *Lolium* closer to *Avena* than to *Triticum* and *Hordeum* (34–37).

The other fraction of prolamins is constituted by glutenins, which are present as high molecular weight (HMW) and low molecular weight (LMW) subunits that join to make multimeric proteins held by disulphide bonds (1). Immune reactive epitopes have also been reported for HMW and LMW glutenins (38–43).

In this study, seven peptides were measured from three glutenin-like protein sequences. None of these peptides have been reported previously in ryegrass (15). Two peptides QQCCQQLAQIPEQSR and SQMLQQSSCHVIR (Figure 3G) from protein J9QGY5 (Gliadin-domain containing protein family) were found exclusively in LMW-GS of the species *D. villosum* of the Triticeae tribe. Both peptides were experimentally detected in ryegrass but not in wheat; the peptide SQMLQQSSCHVIR showed consistent levels across the ryegrass cultivars. Five peptides were characteristic of HMW-GS. These included four peptides (ELQESSLEACR, DVSAKCRPVAVSQVAR, ELQESSLEACRQVVDQQLAGR, and QLQCERELQESSLEACR, Supplementary Figures 1C,D, Figure 4) matching to protein X5CHT6. These were determined to be conserved within the Triticeae tribe and had variable abundance within the analysed ryegrass cultivars. The peptide ELQESSLEACR (Supplementary Figures 1C,D) was highly abundant in the wheat cultivars. Peptide DGSFYPGEATPPQQLQQR was found consistently in ryegrass samples and was not detected in the wheat extracts; it was exclusively found in a protein sequence from *E. libanoticus*, which is a wild species of the Triticeae tribe.

Two peptides characteristic of LMW glutenin (QQCCQQLAQIPEQSR and SQMLQQSSCHVIR) and one peptide characteristic of HMW glutenin (DGSFYPGEATPPQQLQQR) are found within Triticeae tribe; however, they were not present in wheat protein sequences. As such, these sequences may be candidate peptides to differentiate wheat and ryegrass. Nevertheless, this study reports candidate glutenin peptides for the first time in ryegrass cultivars.

#### Tryp_Alpha_Amyl Family

Alpha-amylase/trypsin inhibitor proteins are not considered as gluten proteins; however, recent research suggests that some GRDs are not triggered by gluten proteins but by other types of proteins with similar structures, including ATI proteins (44, 45). These ATI proteins are involved in plant defence; however, they also activate the innate immune system and trigger pro-inflammatory cytokines (46–49). It is believed that ATIs can affect individuals with a sensitive type of GRD (46, 47).

ATI proteins were identified in this study, and diagnostic peptides were measured by LC-MS. The strategic approach consisted of searching peptides characteristic of proteins with the protein family domain PF00234, which lead to the initial recognition of several proteins and peptides; however, a deeper analysis using protein and peptide homology searches revealed that some of these peptides were found in other members of the PF00234-domain containing proteins, namely nsLTPs and puroindolines. Ultimately, four peptides characteristic of ATI proteins were measured from three target proteins. Two peptides (LQCVGSQVPEAVLR and EGMEVFPGCR, Supplementary Figures 1A,B, Figure 5A) from protein T1WIP3 were conserved for species of the Triticeae tribe, which coincides with the quantitative measurements showing a higher abundance in the wheat cultivars. Peptide LTAASVPAVCK (Figure 5C) from the protein T1WI30 had variable abundance across the ryegrass cultivars and was found in sequences from several species of the Triticeae tribe. Consequently, these peptides are unlikely candidates for markers of ryegrass presence. The last peptide, RCCDELSAIPAYCR (Figure 5D), from protein Q84VT9 showed high abundance in ryegrass and was not detected in wheat. This peptide was found exclusively in alpha-amylase/trypsin inhibitor proteins of species *Hordeum vulgare*; therefore, it is not present in wheat databases and is a candidate peptide to differentiate ryegrass from wheat but not barley.

The results described herein for the identification of ATI proteins provide evidence for peptide sequence similarity when comparing ryegrass to protein members of the Triticeae tribe ATIs, but further analysis, supplemented by genomic/transcriptomic sequencing efforts, would be needed to ascertain the extent of protein sequence homology. Principally, the ATIs targeted herein from ryegrass cultivars may not necessarily have the same immunoinflammatory properties as the wheat ATIs that are implicated in GRD. ATIs often differ in secondary structure, i.e., the number and position of intrachain disulphide bonds, and may contain different arrangements of α-helices that can influence the ability to activate the innate immune response (47).

## Conclusion

Ryegrass is a common field contaminant with dense seeds and herbicide-resistant properties. These factors render ryegrass with the potential to enter the supply chain and be inadvertently consumed by the general population. This study provides evidence that gluten-like peptides are present in perennial ryegrass, thereby highlighting the potential risk of unintentional consumption of gluten through the cross-contamination of traditional cereal grains. Indeed, this food safety risk is underscored by a lack of knowledge around the potential for the industry standard ELISA assessment to produce inaccurate information. If these new gluten protein species trigger a Coeliac response but fail to produce an ELISA signal, then a food safety issue may remain unchecked. Conversely, should ryegrass produce no Coeliac response but a strong ELISA signal, then foods may not meet safety requirements in error, thereby presenting an unnecessary challenge for food manufacturers. Future studies are warranted to explore the immunogenic potential of these new gluten-like proteins to determine their presence in nontraditional cereal grains, determine their natural variation, and deploy methods that can be used to differentiate ryegrass from wheat.

Through targeted measurement of prolamin super-family proteins, a suite of peptides were identified that showed consistent abundance across ryegrass cultivars but were not detected in wheat. These peptides could potentially be used in an assay for detecting ryegrass contamination in food products and differentiating ryegrass from wheat contamination in other cereal grains or processed foods. The methodology developed herein could also be applied to determine the extent of ryegrass presence in commodity grain or after processing into food ingredients. There are however no studies that have reported on whether the gluten-like proteins from ryegrass can trigger CD or other GRDs. Nevertheless, one study revealed cross-reactivity between ryegrass pollen and wheat endosperm proteins (50). Future investigation is required to measure the immune reactive potential of ryegrass and continue this important body of work that now spans over 90 years. Importantly, further studies are also required to enhance the genomic resources available for ryegrass so that species-specific proteins are readily identifiable rather than relying on sequence variation from related taxa to identify peptides and proteins. A combination of clinical studies supplemented by analytical workflows to understand the risk associated with agricultural co-mingling and dietary exposure are needed to ensure food safety and avoid the inadvertent failure of the GF-diet.

## Data Availability Statement

The datasets presented in this study can be found in online repositories. The names of the repository/repositories and accession numbers can be found below: https://doi.org/10.25919/8ehe-yr54.

## Author Contributions

SE-C, JB, MC, and CH designed the study. SS and AA carried out sample preparation and extraction. SE-C and JB carried out acquisition, analysis, interpretation of the data and created the scripts for data curation, and visualisation. AJ provided guidance for building the database, data analysis, data visualisation, and data interpretation. SE-C, JB, AJ, CH, and MC drafted the manuscript. All authors contributed to the article and approved the submitted version.

## Conflict of Interest

The authors declare that the research was conducted in the absence of any commercial or financial relationships that could be construed as a potential conflict of interest.

## Publisher's Note

All claims expressed in this article are solely those of the authors and do not necessarily represent those of their affiliated organizations, or those of the publisher, the editors and the reviewers. Any product that may be evaluated in this article, or claim that may be made by its manufacturer, is not guaranteed or endorsed by the publisher.

## Footnotes

^1^ https://github.com/Sophia-006/FDR_proteins_peptides.git

^2^ Uniprot.org

^3^ https://software.broadinstitute.org/morpheus/
