# Supplementary material for: Perennial Ryegrass Contains Gluten-Like Proteins That Could Contaminate Cereal Crops
Source: Front Nutr. 2021 Jul 28;8:708122. doi: 10.3389/fnut.2021.708122 (PMC8355629; doi:10.3389/fnut.2021.708122)
Supplement: Supplementary file 2 [file Data_Sheet_1.docx]

Supplementary Material

# Supplementary Data

Mass spectrometry proteomics data have been deposited in:

<https://doi.org/10.25919/8ehe-yr54>

Supplementary Data Files of the manuscript can be found in the .xlsx

# Supplementary Figures and Tables

## Supplementary Figures


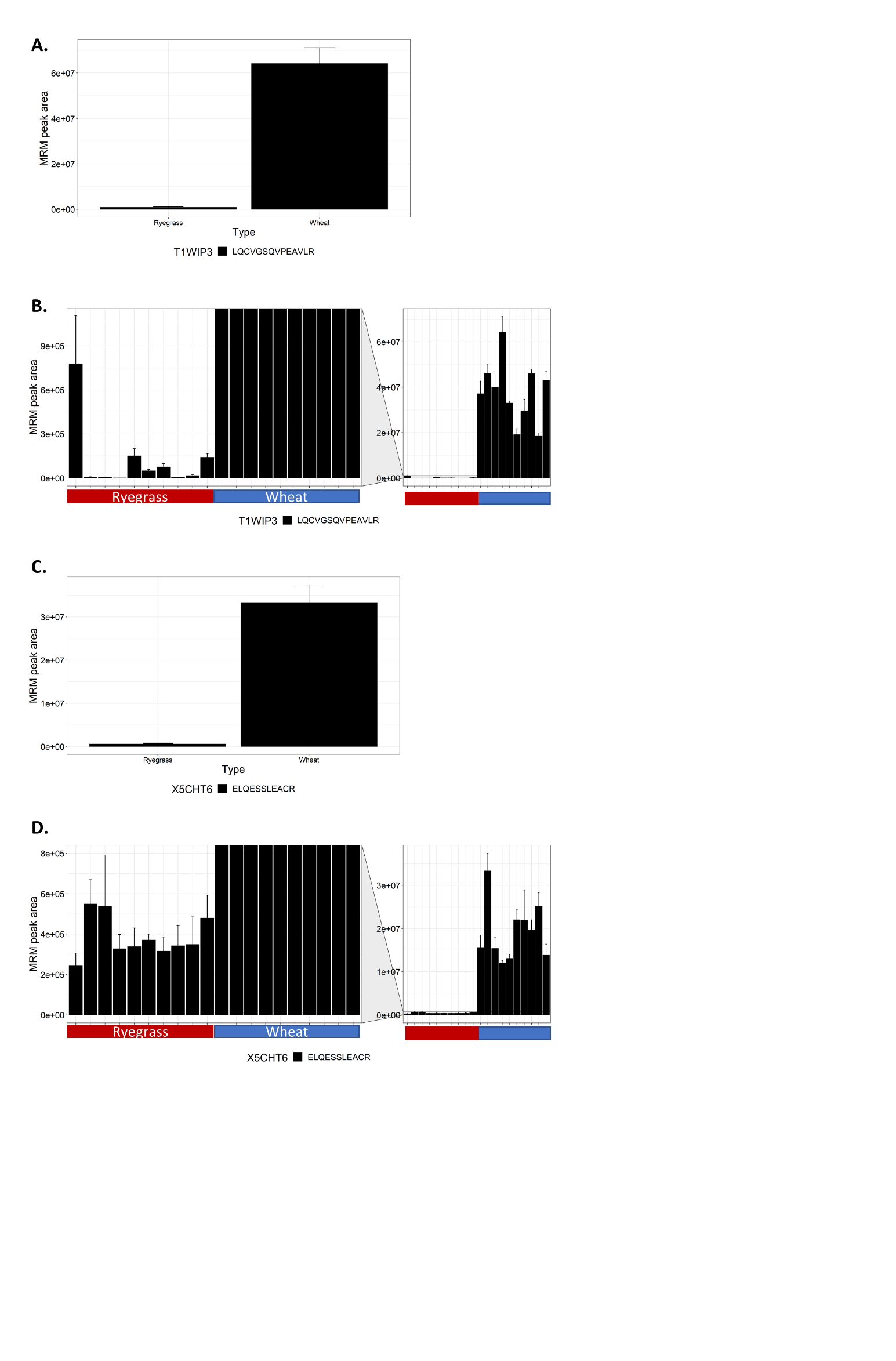
**Supplementary Figure 1.** MRM peak area comparison between ryegrass and wheat for peptides with higher expression in wheat. Data is presented as the mean ± SD (ryegrass n=3, wheat n=4) with one peptide for each protein. 2% percent of overall signal is attributable to ryegrass. Panels A-D refer to the different measured proteins, with their accession specified below each chart, followed by the monitored peptide sequences.

## Supplementary Tables

**Supplementary Table 1**. Ryegrass and wheat cultivars used in this study. Details of species, ploidy, Australian Pastures Genebank (APG) number or Australian identity (AUS), are specified.

| Cultivar | SPECIES | Ploidy | APG/aUS IDENTITY |
| --- | --- | --- | --- |
| Victorian | *Lolium perenne* | Diploid | APG 7898 |
| SF Hustle | *Lolium perenne* | Diploid | APG 83893 |
| Roper | *Lolium perenne* | Diploid | APG 83889 |
| Platinum | *Lolium perenne* | Diploid | APG 83894 |
| Kingston | *Lolium perenne* | Diploid | APG 61239 |
| Expo | *Lolium perenne* | Diploid | APG 83901 |
| Excess | *Lolium perenne* | Diploid | APG 83896 |
| Dobson | *Lolium perenne* | Diploid | APG 61436 |
| Avalon | *Lolium perenne* | Diploid | APG 40099 |
| Ansa | *Lolium perenne* | Diploid | APG 83895 |
| Ventura | *Triticum aestivum* | Hexaploid | AUS 34030 |
| Sunvale | *Triticum aestivum* | Hexaploid | AUS 24930 |
| Magenta | *Triticum aestivum* | Hexaploid | AUS 36469 |
| Mace | *Triticum aestivum* | Hexaploid | AUS 36672 |
| Janz 2 | *Triticum aestivum* | Hexaploid | AUS 24794 |
| EGA Kidman | *Triticum aestivum* | Hexaploid | AUS 36348 |
| EGA Gregory | *Triticum aestivum* | Hexaploid | AUS 34283 |
| EGA Bonnie Rock | *Triticum aestivum* | Hexaploid | AUS 34399 |
| Diamond Bird | *Triticum aestivum* | Hexaploid | AUS 27647 |
| Calingiri | *Triticum aestivum* | Hexaploid | AUS 29490 |

**Supplementary Table 2.** **Peptide search results.** Peptides measured with MRM-MS were submitted to a peptide search using UniProt tool. Peptide sequence, protein accession, and protein family is specified. Protein homology refers to all protein types where the peptide was found. Peptide search results refer to all species where the peptide was found with 100% identity match.

| **Peptide sequence (K/R at the beginning)** | **Protein Accession** | **Protein family** | **Protein homology** | **Peptide search results**  **100% identity match (Species)** |
| --- | --- | --- | --- | --- |
| SQILQQSSCQVMR | G8ZCU8 | PF13016 | Avenin | *Avena canariensis, Avena clauda, Avena damascene, Avena eriantha, Avena insularis, Avena longiglumis, Avena macrostachya, Avena magna, Avena murphyi, Avena prostrata, Avena sativa, Avena ventricose* |
| CPAIHSVVQAIILQK | I4EP61 | PF13016 | Avenin | *Avena canariensis, Avena clauda, Avena damascene, Avena eriantha, Avena insularis, Avena longiglumis, Avena macrostachya, Avena magna, Avena murphyi, Avena prostrata, Avena sativa, Avena strigosa, Avena ventricose* |
| QFLVQQCSPVAEVPFLR | I4EP61 | PF13016 | Avenin | *Avena canariensis, Avena clauda, Avena damascene, Avena eriantha, Avena insularis, Avena longiglumis, Avena macrostachya, Avena magna, Avena murphyi, Avena prostrata, Avena sativa, Avena strigosa, Avena ventricose* |
| AFALQALPAMCDVYVPPHCSVA | I4EP61 | PF13016 | Avenin | *Avena canariensis, Avena damascene, Avena damascene, Avena longiglumis, Avena macrostachya, Avena magna, Avena murphyi, Avena prostrata, Avena sativa, Avena strigosa, Avena* |
| QQAQFEGMR | I4EP57 | PF13016 | Avenin | *Avena murphyi* |
| QQCCQQLAQIPQQLR | F2X0K8 | PF13016 | Gamma-gliadin | *Triticum aestivum, Triticum monococcum* |
| APFASIVASIGGQE | F2X322 | PF13016 | Gamma-gliadin | *Triticum aestivum* |
| APFASIVAGIGGQYR | B6DQD5 | PF13016 | Gamma-gliadin | *Aegilops tauschii, Triticum aestivum, Triticum turgidum* |
| QQCCQQLAQIPEQSR | J9QGY5 | PF13016 | Low molecular weight glutenin | *Dasypyrum villosum* |
| SQMLQQSSCHVIR | J9QGY5 | PF13016 | Low molecular weight glutenin | *Dasypyrum villosum* |
| DVSAKCRPVAVSQVAR | X5CHT6 | Glutenin | High molecular weight glutenin | *Aegilops comosa, Aegilops cylindrica, Aegilops geniculata, Aegilops kotschyi, Aegilops longissima, Aegilops markgrafii, Aegilops sharonensis, Aegilops speltoides, Aegilops uniaristata, Thinopyrum elongatum, Thinopyrum intermedium, Triticum aestivum, Triticum dicoccoides, Triticum monococcum, Triticum timopheevii* |
| ELQESSLEACRQVVDQQLAGR | X5CHT6 | Glutenin | High molecular weight glutenin | *Aegilops bicornis, Aegilops comosa, Aegilops cylindrica, Aegilops longissimi, Aegilops markgrafii, Aegilops searsii, Aegilops sharonensis, Aegilops speltoides, Aegilops tauschii, Aegilops umbellulate, Aegilops ventricose, Secale cereale, Taeniatherum caput-medusae, Thinopyrum elongatum, Triticum aestivum, Triticum compactum, Triticum dicoccoides, Triticum dicoccon, Triticum monococcum, Triticum spelta, Triticum timopheevii, Triticum turgidum* |
| ELQESSLEACR | X5CHT6 | Glutenin | High molecular weight glutenin | *Aegilops bicornis, Aegilops comosa, Aegilops cylindrica, Aegilops geniculata, Aegilops kotschyi, Aegilops longissima, Aegilops markgrafii, Aegilops searsii, Aegilops sharonensis, Aegilops speltoides, Aegilops tauschii, egilops umbellulate, Aegilops uniaristata, Aegilops ventricose, Agropyron cristatum, Australopyrum retrofractum, Dasypyrum hordeaceum, Dasypyrum villosum, Elymus alashanicus, Elymus canadensis, Elymus ciliaris, Elymus libanoticus, Elymus libanoticus, Elymus nakaii, Elymus sibiricus, Elymus stipifolius, Eremopyrum bonaepartis, Eremopyrum triticeum, Hordeum vulgare, Leymus mollis, Leymus multicaulis, Leymus racemosus, Psathyrostachys juncea, Pseudoroegneria spicata, Pseudoroegneria strigose, Secale cereale, Thinopyrum elongatum, Thinopyrum intermedium, Thinopyrum junceum, Triticum aestivum, Triticum compactum, Triticum dicoccoides, Triticum dicoccon, Triticum monococcum, Triticum spelta, Triticum timopheevii, Triticum Urartu* |
| QLQCERELQESSLEACR | X5CHT6 | Glutenin | High molecular weight glutenin | *Aegilops bicornis, Aegilops comosa, Aegilops cylindrica, Aegilops geniculata, Aegilops kotschyi, Aegilops longissima, Aegilops markgrafii, Aegilops searsii, Aegilops sharonensis, Aegilops speltoides, Aegilops tauschii, Aegilops umbellulate, Aegilops uniaristata, Aegilops ventricose, Agropyron cristatum, Hordeum vulgare, Psathyrostachys juncea, Secale cereale, Thinopyrum elongatum, Thinopyrum intermedium, Thinopyrum junceum, Triticum aestivum, Triticum compactum, Triticum dicoccoides, Triticum dicoccon, Triticum monococcum, Triticum spelta, Triticum timopheevii, Triticum turgidum, Triticum urartu* |
| DGSFYPGEATPPQQLQQR | H6UQP6 | Glutenin | High molecular weight glutenin | *Elymus libanoticus* |
| RCCDELSAIPAYCR | Q84VT9 | PF00234 | Trypsin inhibitor | *Hordeum vulgare* |
| LQCVGSQVPEAVLR | T1WIP3 | PF00234 | Dimeric alpha-amylase inhibitor | *Aegilops bicornis, Aegilops longissimi, Aegilops tauschii, Elymus brevipes, Elymus caucasicus, Elymus ciliaris, Elymus grandis, Elymus tibeticus, Elymus pilifer, Kengyilia alatavica, Kengyilia grandiglumis, Kengyilia kokonorica, Kengyilia melanthera, Kengyilia mutica, Kengyilia rigidula, Kengyilia thoroldiana, Pseudoroegneria spicata, Pseudoroegneria strigose, Triticum dicoccoides, Triticum monococcum, Triticum spelta, Triticum turgidum, Triticum urartu* |
| EGMEVFPGCR | T1WIP3 | PF00234 | Dimeric alpha-amylase inhibitor | *Elymus grandis* |
| LLQQQLNPCR | A8VZG4 | PF00234 | Alpha-gliadin | *Dasypyrum hordeaceum* |
| LTAASVPAVCK | T1WI30 | PF00234 | Dimeric alpha-amylase inhibitor | *Agropyron cristatum, Agropyron desertorum, Agropyron mongolicum, Douglasdeweya deweyi, Douglasdeweya wangii, Elymus brevipes, Elymus caucasicus, Elymus shandongensis, Eremopyrum bonaepartis, Kengyilia alatavica, Kengyilia batalinii, Kengyilia kaschgarica, Kengyilia tahelacana, Kengyilia zhaosuensis, Triticum aestivum* |
| TACNCLK | A0A0P0Y6A6 | PF00234 | Non-specific lipid-transfer protein | *Very conserved. Families: Poaceae, Fabaceae, Asteraceae* |
| CGVSIPYTISPSIDCSR | A0A0P0Y6A6 | PF00234 | Non-specific lipid-transfer protein | *Oryza barthii, Oryza sativa* |
| DPYYEQCPMRK | C3TX90 | PF00234 | Puroindoline-like protein | *Brachypodium sylvaticum* |
| SDLYGPNLQGEVTMLMER | C3TX90 | PF00234 | Puroindoline-like protein | *Brachypodium sylvaticum* |
| QLSQIAPQCR | D7FSI5 | PF00234 | Puroindoline, Hordoindoline | *Aegilops comosa, Aegilops kotschyi, Aegilops longissimi, Aegilops markgrafii, Aegilops searsii, Aegilops sharonensis, Aegilops speltoides, Aegilops tauschii, Aegilops umbellulate, Aegilops uniaristata, Aegilops ventricose, Agropyron cristatum, Australopyrum retrofractum, Avena insularis, Avena sativa, Bromus inermis, Dasypyrum villosum, Elymus burchan-buddae, Elymus excelsus, Elymus libanoticus, Elymus nutans, Eremopyrum bonaepartis, Henrardia persica, Psathyrostachys huashanica, Psathyrostachys juncea, Taeniatherum caput-medusae, Triticum aestivum, Triticum compactum, Triticum macha, Triticum monococcum* |
